# Cervical epidural abscess due to implantation of a spinal cord stimulation lead

**DOI:** 10.1002/ccr3.5931

**Published:** 2022-06-02

**Authors:** Gregor A. Bara, Jost Thissen

**Affiliations:** ^1^ 9374 Department of Neurosurgery University of Bonn Bonn Germany; ^2^ Spine Center Schoen Clinic Düsseldorf Germany

**Keywords:** implant infection, spinal cord stimulation, spinal epidural abscess

## Abstract

Spinal cord stimulation (SCS) for intractable pain syndromes has become a pillar of modern pain management. Common complications include lead migration, implant infection, cerebral spinal fluid leak, and lead fracture. Spinal epidural abscess due to spinal cord stimulator implantation is a very rare occurrence with only two cases reported in the literature so far. We present an illustrative case and discuss the pathophysiology and best clinical management for this very rate entity.

## Graphical abstract

Surgical site infections are a serious adverse event of neurological implant surgery. For prevention, strict adherence to existing guidelines is advised. In case of deep infection, all previously implanted hardware should be removed and antibiotic therapy started. Upon suspicion of deep infection, especially a spinal epidural abscess, MR imaging of the neuroaxis should be performed. Further surgical management may be necessary, that is, drainage of the epidural abscess.

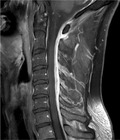

## 1 INTRODUCTION

Spinal cord stimulation (SCS) for intractable pain syndromes has become a pillar of modern pain management. It has proven to provide significant long‐term pain reduction as well as improvement of quality of life.^1^ SCS is considered to be a safe technique with a risk profile mainly consisting of lead migration, implant infection, cerebral spinal fluid leak, lead fracture, and discomfort at the site of the internal pulse generator. However, spinal epidural hematomas have been reported as well.^2^ Spinal epidural abscess due to spinal cord stimulator implantation is a very rare occurrence. Only two cases have been reported in the literature so far.^3^, ^4^

We present the case of a spinal epidural abscess after implantation of spinal cord stimulation leads to presenting with gait ataxia. We discuss the pathophysiology and best clinical management for this very rate entity.

## 2 CASE PRESENTATION

**FIGURE 1:**
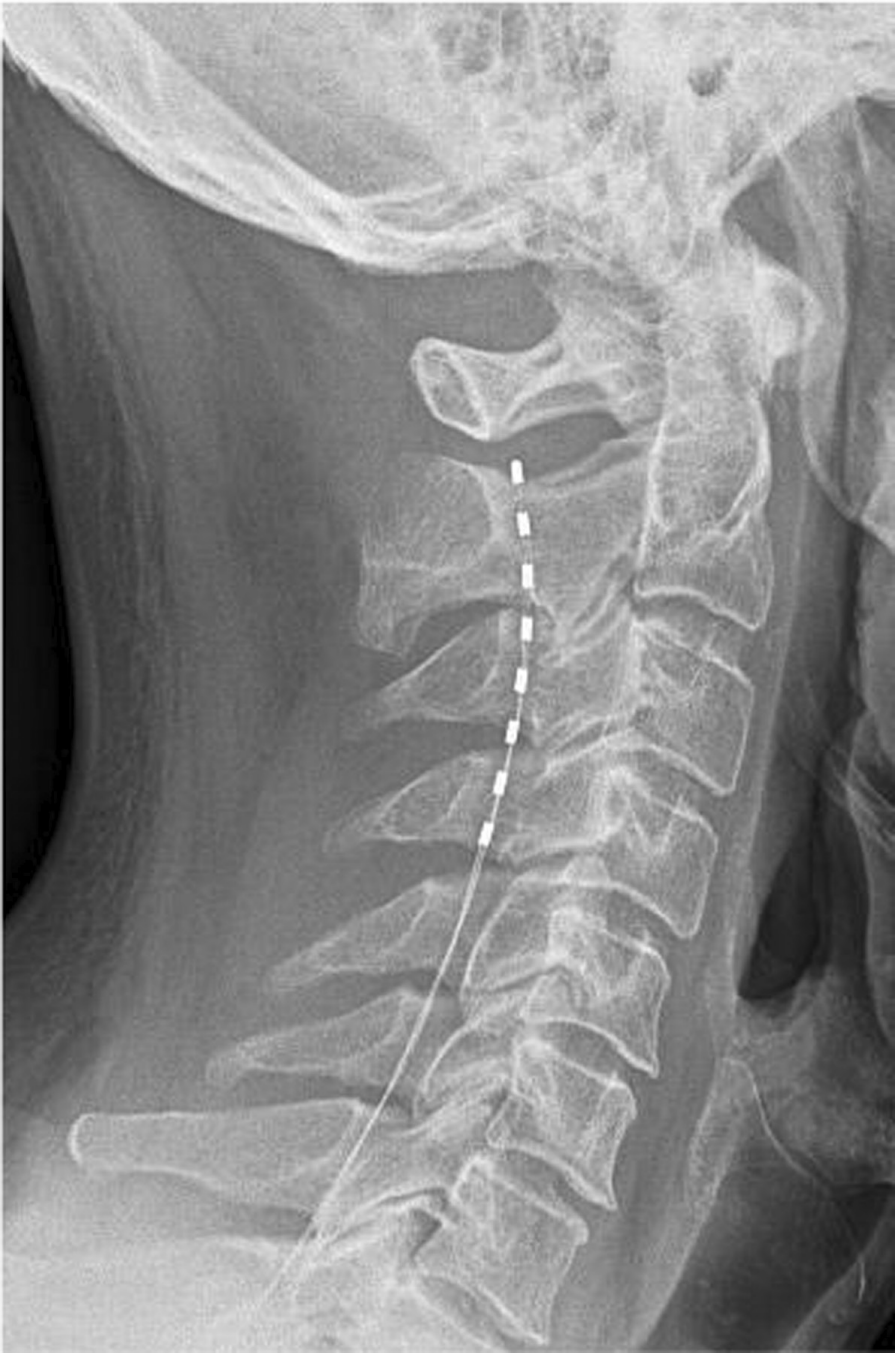
X‐Ray of the cervical spine showing the lead position

**FIGURE 2:**
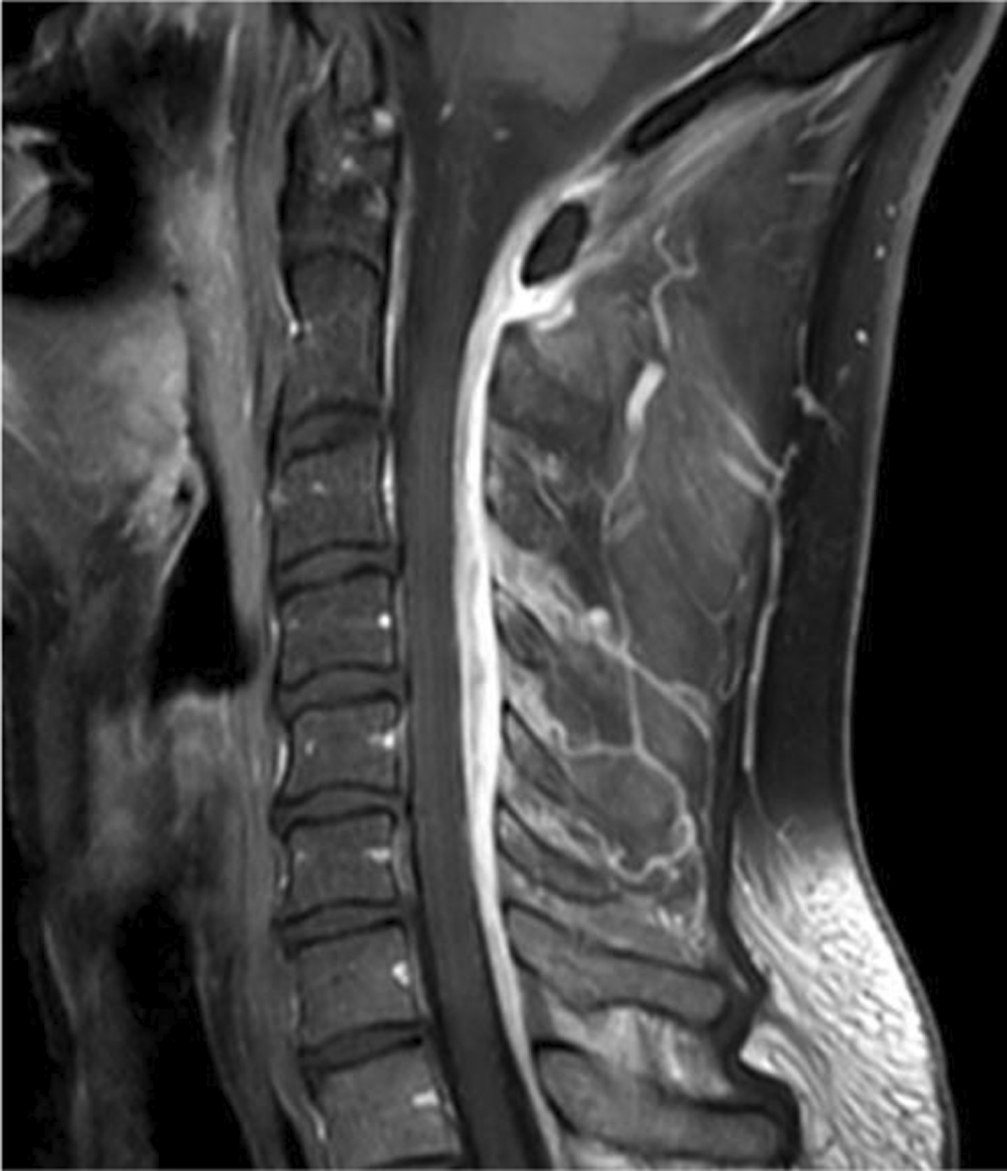
MRI scan in T1 sequence with gadolinium contrast agent showing dorsal epidural enhancement consistent with an epidural abscess

A 49‐year‐old man was treated for chronic back and neck pain which he was suffering since his adolescence. He had previously undergone multimodal conventional medical management with metamizole, etoricoxib, pregabalin, oxycodon, and amitriptyline as well as physiotherapy and psychotherapy. MRI scans showed no signs of nerval compression. Therefore, we indicated a trial for spinal cord stimulation. The patient's medical history did not reveal any other comorbidities despite chronic pain, in particular, there were no risk factors regarding a possible infection (no tobacco use, no hyperglycemia/diabetes, no malignancy, no malnutrition, no HIV infection, no steroid use, no remote infection, no active malignancy, no use of anticoagulants). Physical examination did not show any skin abnormalities. The day before surgery, patients are admitted to the hospital and receive the standard protocol anesthesia work‐up, electrocardiogram (ECG), screening for MSSA/MRSA, and laboratory test. There were no pathological findings. Surgery was performed in the operating room of the neurosurgery unit under local anesthesia. The patient was positioned prone on the operating table. For preoperative skin preparation, local hair was removed with an electrical clipper followed by application of Octeniderm^®^ (Schülke & Mayr GmbH, Norderstedt, Germany. Composition of 100 g solution: octenidine dihydrochloride 0.1 g, 1‐propanol 30.0 g, 2‐propanol 45.0 g) to the entire relevant surgical field. Aseptic draping of the surgical field followed by an impregnated plastic adhesive drape was applied. The procedure was performed by an experienced surgeon regarding neuromodulation procedures with a history of more than 1000 implants with adequate surgical scrub and surgical attire. Local anesthetic solution (xylocaine 2% with adrenaline 1:200 000) was administered to minimize postoperative pain and prevent bleeding. A three‐centimeter‐long incision was performed lumbar and a single spinal cord stimulation lead (octrode 90 cm, Abbott) was introduced into the epidural space and advanced further cephalad until reaching the level of C2 (see Figure 1). A lateral view confirmed dorsal placement. An intraoperative test stimulation verified paresthesia overlapped with the patient's experienced pain. The lead was anchored to the lumbar fascia with an anchoring device (swift lock, Abbott) and connected to an extension lead which was externalized circa ten centimeters laterally to the initial skin incision. The skin incision was then irrigated with saline and closed firmly with subcutaneous and cutaneous sutures. The extension lead was anchored to the skin with a purse‐string stitch and sterile dressings were applied to all surgical wounds. The extension lead was then connected to an external pulse generator. From initial incision to final wound closure, the whole surgical procedure lasted 27 min and was un‐eventful. We initially trialed with burst stimulation. Over the course of the next 3 days, the patient experienced pain relief of 50%. Thereafter, pain reduction diminished again until the patient experienced no effect. Seven days after initial implantation, we measured high lead resistances on all channels (>10 000 Ohm), no tonic stimulation could be applied. The trial was, therefore, discontinued as we thought of an early lead breakage and a revision operation was offered. The extension lead was cut on skin level. All wounds did not show any signs of infection. However, the patient did not want to continue with the neuromodulation therapy and an explantation was scheduled. Before the scheduled operation and 3 weeks after the initial implantation of leads, the patient presented in our emergency department. He had lost balance and fallen on the ground. Thereafter, he experienced a sudden increase in neck pain. At no point did the patient show signs of fever or local signs of infection. Laboratory controls did not show an increase in infection parameters. An initial X‐Ray of the cervical spine showed no pathology with the lead still in place. Under analgetic therapy, the pain did not resolve over the next days. We, therefore, explanted the lead to perform an MRI scan of the cervical spine. The operation site did not show any signs of infection. The postoperatively performed MRI scan showed an epidural liquid formation absorbing contrast agent, which was consistent with the picture of an epidural abscess (see Figure 2). We explanted all previously implanted material and performed a left‐sided microsurgical decompression on level C4/5 and discovered the expected epidural abscess. Samples were acquired and sent to microbiology for further analysis. A small catheter was advanced cranially and caudally and the abscess was drained under subtile isotonic fluid insufflation. Calculated intravenous antibiotic therapy was begun after sample acquisition consisting of flucloxacillin, ceftriaxone, and metronidazole. The microbiological analysis identified cutibacterium acnes as cause of the epidural abscess. According to the antibiogram, the antibiotic therapy was switched to amoxicillin/clavulinic acid and administered for a total of 6 weeks. The patient recovered with no neurological deficit.

## 3 DISCUSSION

Implant device‐related surgical site infections (SSI) are defined as such infections occurring within 1 year postoperatively at the site of an implanted device and are associated with an increase in morbidity and mortality as well as expanding healthcare costs. Three major types of surgical site infections are superficial infections, deep infections with involvement of the lead and/or the internal pulse generator, and epidural abscess.^5^

Infection rates of neuromodulation devices seem to be higher than other implanted devices (such as pacemakers or joint replacements).^6^ Two systematic reviews have reported infection rates of 3.4%–4.6%.^7^, ^8^

The majority of deep infections occur at the implantation site of the internal pulse generator (54%), followed by the implantation site of the lead (17%). The most common causative agents are Staphylococcus species (48%).^9^

The Neuromodulation Appropriateness Consensus Committee has developed recommendations for infection prevention and management regarding the preoperative work‐up, the surgical procedure itself as well as the postoperative care.^5^

The preoperative work‐up consists of acquirement of the patient's medical history as well as optimizing medical comorbidities. Tobacco use,^10^, ^11^ hyperglycemia, or uncontrolled diabetes,^12^ obesity, active malignancy and currently undergoing chemotherapy, human immunodeficiency virus infection with high viral load (30 000 copies per ml or more),^13^ untreated remote infections,^14^ preoperative steroid use,^15^ *S. aureus* carriers, anticoagulant use,^16^ opioid use,^17^ malnutrition, immunosuppressant intake^18^ as well as radiation therapy^19^ have been identified as risk factors. Therefore, optimization of diabetic management, smoking cessation, limiting steroid, treatment of potential infection sources, optimization of nutritional status, optimization of HI viral load, as well as consultation with oncology regarding risk stratification are advised. As the presence of hematoma constitutes a risk for wound dehiscence and provides a bacterial growth medium, an appropriate management of the anticoagulation therapy should be considered. The physical examination should be undertaken carefully to rule out local skin infections or skin abnormalities. Measurement of vital signs and laboratory evaluation may provide further hints for systemic infections (increased temperature, heart rate, and blood pressure, increased white blood cell count, erythrocyte sedimentation rate, and level of C‐reactive protein). Staphylococcus aureus colonized patients should be decolonized.^5^

Prior to surgery, local hair should be removed with electrical clippers, preoperative weight‐based dosed antibiotics should be administered. Skin preparation with chlorhexidine‐based products combined with isopropyl alcohol is recommended. If adhesive drapes are used, they should be iodophor‐impregnated drapes. Physicians should perform a preoperative surgical scrub for a minimum of 2–5 min and maximal sterile barrier precautions including double gloving are recommended. The operating staff should minimize traffic flow through the operating room. Further, usage of sterile C‐arm drapes, minimization of contact with overhead lights, and the C‐arm drape are recommended. As surgical training may limit infections, all procedures should be performed by implanters who were trained under supervision with a minimum of ten cases as the primary implanter. The surgical technique should include an appropriate intraoperative tissue management and limit surgical tissue trauma. Before wound closure, an irrigation with saline through a bulb syringe should be performed. Wound closure should be performed appropriate.^5^

For postoperative wound care, sterile occlusive dressings for 24–48 h are recommended. While preoperative administration of antibiotics has shown to be beneficial, prolonged postoperative antibiotics have not shown to improve outcomes and are, therefore, not recommended. However, in case of high‐risk patients, this should be considered. Upon discharge, patients should be appropriately educated on signs and symptoms of infection. In case of occurrence of an infection, an infectious disease specialist should be consulted to refine the treatment, in case of suspicion of deep wound infections including epidural involvement MR imagining of the neuroaxis is recommended. A re‐implantation should be considered after finalized treatment of the infection.^5^

This case report demonstrates that a large cervical abscess may occur due to spinal cord stimulation lead implantation and may be treated by appropriate and early surgery. Further, sudden increase in lead resistance might be an early sign of abscess formation around the lead. In this case, apart from gait ataxia (as a new neurological symptom), no other clinical signs of infection could be found. The operation was performed under strict aseptic condition with standard peri‐procedural antibiotics following all NACC recommendations. Postoperative care consisted of proper wound care and dressings. This young patient did not have a history of infections, had no predisposing comorbidities (no tobacco use, no hyperglycemia/diabetes, no malignancy, no malnutrition, no HIV infection, no steroid use, no remote infection, no active malignancy, no use of anticoagulants) and did not show any pathological findings during the initial preoperative work‐up.

Spinal epidural abscess is a very rare incident defined as a severe, generally pyogenic infection of the epidural space. This condition is associated with a poor outcome requiring imminent neurosurgical intervention to avoid permanent neurologic deficits. The gold standard of diagnosis is magnetic resonance imaging with contrast agent. Although some cases have been reported to be fungal, most cases are bacterial and attributed to hematogenic spread, one third occurs via continuous spread such as osteomyelitis, discitis, or paraspinal abscess. The most common etiological agent is staphylococcus aureus. Coagulase‐negative staphylococcus occurs much rarer. Gram‐negative bacteria may occur in drug abusers or immune‐deprived patients. In general, risk factors are older age, diabetes mellitus, immune system disorders, prior hospitalization, malnutrition, obesity, and smoking. An epidural abscess is hard to diagnose due to its wide range of symptoms. Axial pain and fever are the most common; however, a wide range of neurological symptoms such as hypesthesia, motor deficit, ataxia, or in the worst case, paraplegia may occur.^20^ The underlying pathophysiology includes mechanical compression of the spinal cord by the expanding abscess, spinal vessel thrombosis, and impaired spinal cord perfusion.^21^ We presume that the loss of clinical effect of burst stimulation, the inability to perform tonic stimulation, as well as the impedance change have been due to the expanding abscess. The gold standard for management of spinal epidural abscess presenting with neurological deficit is considered to be surgical decompression. Various studies have shown surgical management to be superior to conservative management and to be associated with improvement in neurological status. Surgical management depends of localization of the abscess (cervical, thoracic, lumbar, anterior, posterior) as well as the involvement of disks and vertebral bodies and may include posterior decompression via laminotomy or (hemi)‐laminectomy, fusion as well as corporectomy.^22^, ^23^

Regarding the mechanism of infection, it is most likely that infection occurred during the time of the implant given the swift dynamic of the infection although a hematogenous spread from an unrelated (yet in this case clinically undiagnosed unrelated infection) may be another seeding pathway that can result in device infection. As cutibacterium acnes is considered a cutaneous bacterium, an intra‐ or peri‐operative mechanism of infection becomes more likely. Possible mechanism of introduction of the bacterium may be contact with the lead by the implanter (despite double gloving which should reduce the number of inner glove perforations), contact with the lead by the patient's skin (despite impregnated plastic adhesive drape), or continuous growth of patients skin flora at the incision site as well as the extension site (despite adequate skin closure). Given the sole finding of a cervical epidural abscess without any local signs of infection on skin level, the introduction of the bacterium via the tip of the lead becomes most likely. The development of the deep infection may have been further supported by an epidural vein hemorrhage which serves as a growing medium for introduced bacteria. However, this may be unlikely as the patient did not take any anticoagulants and an epidural hemorrhage would have made neurological symptoms straight after implantation.

Given all these findings, we want to underline the value of double gloving and usage of impregnated plastic adhesive drapes. Further, all implanted material should be touched as little as possible.

## 4 CONCLUSION

This case report serves as a reminder that even a presumably simple implantation of a cylindrical lead for spinal cord stimulation under strict aseptic conditions can cause severe adverse effects. It should alert us that there is no such thing as a simple operation. Spinal epidural abscess can have an incidious clinical presentation with severe neurological deficit. Standard treatment includes surgical drainage of abscess followed by antibiotic treatment.

## AUTHOR CONTRIBUTIONS

Gregor Bara wrote the manuscript. Jost Thissen involved in proof reading.

## CONFLICT OF INTEREST

The authors report no conflicts of interest. The authors alone are responsible for the content and writing of this work.

## ETHICAL APPROVAL

All procedures performed were in accordance with the ethical standards of the institutional and national research committee (Ethic committee of the Rheinische Friedrich Wilhelms University Bonn) and with the 1964 Helsinki declaration and its later amendments or comparable ethical standards.

## CONSENT

The author transfers to the journal the non‐exclusive publication rights and he warrants that his contribution is original and that he has full power to make this grant. The author signs for and accepts responsibility for releasing this material on behalf of any and all co‐authors. This transfer of publication rights covers the non‐exclusive right to reproduce and distribute the article, including reprints, translations, photographic reproductions, microform, electronic form (offline, online), or any other reproductions of similar nature.

## Data Availability

All data generated or analyzed during this study are included in this published article.
